# Digital Language Death

**DOI:** 10.1371/journal.pone.0077056

**Published:** 2013-10-22

**Authors:** András Kornai

**Affiliations:** Computer and Automation Research Institute, Hungarian Academy of Sciences, Budapest, Hungary; Max Planck Institute for the Physics of Complex Systems, Germany

## Abstract

Of the approximately 7,000 languages spoken today, some 2,500 are generally considered endangered. Here we argue that this consensus figure vastly underestimates the danger of digital language death, in that less than 5% of all languages can still ascend to the digital realm. We present evidence of a massive die-off caused by the digital divide.

## Introduction

The biological metaphor of viewing languages as long-lived organisms goes back at least to Herder [1], and has been clearly stated in *The Descent of Man* [2]:

The formation of different languages and of distinct species, and the proofs that both have been developed through a gradual process, are curiously parallel. (…) We find in distinct languages striking homologies due to community of descent, and analogies due to a similar process of formation. The manner in which certain letters or sounds change when others change is very like correlated growth. (…) Languages, like organic beings, can be classed in groups under groups; and they can be classed either naturally according to descent, or artificially by other characters. Dominant languages and dialects spread widely, and lead to the gradual extinction of other tongues.

While not without its detractors [3], the biological metaphor has been widely accepted both in research concerning language death [4],[5] and in guiding political action (see e.g. the United Nations Environment Programme Convention on Biological Diversity,[6]). Here we investigate the phenomenon of *digital ascent* whereby languages enter the space of digitally mediated communication. We could extend the metaphor and talk about the digital *hatching, pupation,* or *metamorphosis* of languages, but would gain little by doing so, since we can only speculate about further, post-digital stages in the life cycle of languages.

In this paper, we bring the traditional methods of language vitality assessment to the digital realm. First we transfer the criteria themselves: instead of speaker population we look at the online population, instead of vigorous oral use we look at vigorous online use, and so forth, see Background (i)–(v). Second, we collect data from online sources that reveal the relevant variables or at least provide acceptable proxies for these, see Materials. Third, we introduce a four-way classification into digitally thriving (T), vital (V), heritage (H), and still (S) languages, roughly corresponding to the amount of digital communication that takes place in the language, and manually select prototypical seeds for these classes, see Methods. Finally, multinomial logistic classifiers are built on the seeds and are applied to the rest of the data, see Results. This four-stage method is shown to be robust, and remarkably independent of the manual choice of seeds, see Discussion. The Conclusions section interprets our main result, that the vast majority of the language population, over 8,000 languages, are digitally still, that is, no longer capable of digital ascent.

## Background

A language may not be completely dead until the death of its last speaker, but there are three clear signs of imminent death observable well in advance. First, there is *loss of function,* seen whenever other languages take over entire functional areas such as commerce. Next, there is *loss of prestige*, especially clearly reflected in the attitudes of the younger generation. Finally, there is *loss of competence*, manifested by the emergence of ‘semi-speakers’ who still understand the older generation, but adopt a drastically simplified (reanalyzed) version of the grammar. The phenomenon has been extensively documented e.g. in Menomini [7], Gaelic [8], and Dyrbal [9].

In the digital age, these signs of incipient language death take on the following characteristics. Loss of function *performed digitally* increasingly touches every functional area from day to day communication (texting, email) to commerce, official business, and so on. Loss of prestige is clearly seen in the adage *If it’s not on the web, it does not exist*, and loss of competence boils down to the ability of raising digital natives [10] in your own language. Digital ascent is the opposite process, whereby a language increasingly acquires digital functions and prestige as its speakers increasingly acquire digital skills.

Language endangerment and language death, in the traditional sense, are widely investigated and actively combated phenomena. The modern EGIDS classification [11] extends the Graded Intergenerational Disruption Scale (GIDS) of Fishman [12] to the following 13 categories: 0. International; 1. National; 2 Provincial; 3 Wider communication; 4 Educational; 5 Developing; 6a Vigorous; 6b Threatened; 7 Shifting; 8a Moribund; 8b Nearly Extinct; 9 Dormant; 10 Extinct. Categories 7–8b are considered endangered in the UNESCO Atlas of the World’s Languages in Danger [13], and categories 9–10 are considered extinct. Since these comprise only 17% of the world’s languages, with another 20% (category 6b) vulnerable, one may get the impression that the remaining 63% (these numbers are from [14]) of the world’s languages are more or less in good shape. While this may be true in the traditional sense, the main finding of our paper will be that the vast majority (over 95%) of languages have already lost the capacity to ascend digitally.

Since digital(ized) data persists long after the last speaker is gone, we cannot simply equate failure to ascend with lack of online data. We will make a distinction between digital *heritage* status, where material is available for research and documentation purposes, but the language is not used by native speakers (L1) for communication in the digital world, and digitally *still* status, characterized by lack of even foreign user (L2) digital presence. It is of course very important to move languages from the still to the heritage stage, and there are significant efforts under way to bring data and metadata about languages online and to make both lexical resources and primary texts web-accessible, see the Materials section for an introduction to these. In the Results section we will see that such efforts, laudable as they are, actually contribute very little to the digital vitality of endangered languages. Just as the dodo is no less extinct for skeleta, drawings, or fossils being preserved in museums of natural history, online audio files of an elder tribesman reciting folk poetry will not facilitate digital ascent, and both still and heritage languages are digitally dead in the obvious sense of not serving the communication needs of a language community.

Digital ascent is a relatively new phenomenon, especially on the hundred year timescale common in studies of language death. Digital communication was not an important arena of language functionality until the spread of electronic document creation in the 1970s; the internet and email in the 1980s; the web and blogging in the 1990s; wikis and text messaging (SMS) in the 2000s. Our approach will nonetheless be conservative inasmuch as we simply adopt the standard conceptual framework, and the standard yardsticks, to the digital domain. We will also try to be maximally conservative in the sense that we will interpret the evidence favorably wherever we can, so as to minimize false alarms. There are five confluent factors we consider: (i) the size and demographic composition of the language community; (ii) the prestige of the language; (iii) the identity function of the language; (iv) the level of software support; and (v) wikipedia. The last two may superficially look peculiar to the digital domain, but as we shall see, they are just convenient proxies for assessing a traditional yardstick, the functional spread of the language.

### (i) Community size

The primary traditional measure of vitality is the size and generational composition of the language community. In the digital realm, what we are interested in is the number of digital natives in the language. Since the phenomenon is new, the demographics are highly favorable: once the language community starts creating content by sending text messages, writing blogs, and building wikis, we can reasonably expect that the younger generation will follow suit, especially as digital fora like Facebook are increasingly becoming a means for parents and grandparents to stay in touch with their children. Therefore, we need to assess only the size of the wired community separately, and can assume its demographic composition to be uniformly good.

State censuses generally address the question of linguistic and national identity, and tribe sizes are well known within the community, so it is generally not hard to get at least a rough order of magnitude estimate on the number of speakers. However, in and of itself a large and sustainable population cannot guarantee digital ascent – what we need to consider is population *actively engaged in digitally mediated interaction*. Passive consumption of digital material, especially digital material in an encroaching language, is irrelevant, if not actively harmful to the survival of a threatened language. Michael Krauss’ famous remark “Television is a cultural nerve gas…odorless, painless, tasteless. And deadly.” [15] applies to the web just as well.

Since neither the size of the digitally enabled population nor the digital suitability/prestige of the language are measured by censuses or other regular surveys, we must resort to proxies in assessing digital vitality. The real issue is *the amount of digitally mediated communication that takes place* in the language. Ideally, we should capture all videoconference (Skype), cellphone, Twitter, Facebook, etc. communication and measure the proportion of material in the language in question. Modern language technology has already solved the problem of language identification, the Crúbadán Project [16] actually builds such software for each language. As this technology in no way relies on understanding the contents, privacy concerns are minimized and the barriers to the direct measurement of digital language vitality are primarily organizational: we need to put safeguards in place to make sure that the data will be anonymized, that the people whose communications are monitored give their permission, and so forth. Until such a comprehensive study is conducted, we must use *the publicly available textual material* as our proxy – this has the advantage that all such material was put there knowingly by their authors, so concerns of privacy are resolved in advance. The size of online holdings (excluding wikipedia, see (v) below) was assessed by web crawling. Our methods are described in [17], and some of the results are made available for public download at http://hlt.sztaki.hu/resources/webcorpora.html.

### (ii) Prestige

The second most important measure of vitality is prestige. Since digital communication is universally viewed as more prestigious than communication by traditional means, the intergenerational disruption actually acts in favor of digital ascent, provided the new generation has both the digital means and the interest in language use. In digitally vital languages this happens quite effortlessly and automatically, but languages the new generation no longer considers cool are caught in a pincer movement, with the old generation unable and unwilling to enter the digital world and the younger generation no longer considering the old language relevant. They may not be semi-speakers in the technical sense, as they retain full control over the grammar and vocabulary, but at the same time they may consider the language inappropriate for dealing with the digital realm. An almost laboratory pure example is provided by the two officially recognized varieties of Norwegian, Bokmål and Nynorsk. For many years, the two wikipedias were of roughly equal size, and the best estimates [18] put the proportion of language users at 7∶1. By now, the Bokmå l wikipedia is four times the size of the Nynorsk wikipedia, but Nynorsk is still in the top 50. With a sizeable population of speakers that enjoy a high standard of living, a nearly saturated personal computer market, and good access to broadband networks, based solely on census data and wikipedia statistics Nynorsk would appear a prime candidate for digital ascent. Yet crawling the.no domain demonstrates a striking disparity: we could find 1,620 m words (tokens) of Bokmål but only 26 m words in Nynorsk. Considering that official (government and local government) pages are published in both varieties, the actual proportion of user-generated Nynorsk content is well under 1%. In spite of a finely balanced official language policy propping up Nynorsk, the Norwegian population has already voted with their blogs and tweets to take only Bokmå l with them to the digital age.

The same phenomenon can be seen at the other side of the digital divide. As an example consider Mandinka, which is, besides Swahili, perhaps the single best known African language for the larger American audience, thanks to Alex Hailey’s *Roots*. With 1.35 m speakers, and official status in two countries (Senegal and The Gambia), Mandinka is neither endangered nor threatened in the traditional sense – SIL puts its EGIDS rating at 5 (developing) and notes the positive attitude speakers of all ages have toward the language. However, its failure to digitally ascend appears a foregone conclusion: literacy in the language is below 1%, and the wikipedia incubator [19] has not attracted a single native speaker.

### (iii) Identity function

As we will primarily rely on written material, particular care needs to be taken to distinguish *passive* (read only) web presence such as lexicons, classical literature, or news services, from *active* use in a broad variety of two-way contexts such as social networks, business/commerce, live literature, etc. Language is for communication, and passive presence indicates only efforts at preservation, often by scholars actually outside the language community, not digital vitality. As an example consider Classical Chinese, a language with a sizeable wikipedia, nearly 3,000 articles, and a remarkable user community of over 30,000 L2 users. There are also significant text holdings elsewhere (see in particular http://ctext.org). At the same time, the top-level question in [11], which probes the identity function of a language clearly puts Classical Chinese in the Historical/Heritage category, there defined as follows:

#### Historical

The language has no remaining speakers and no community which associates itself with the language as a language of identity. There are no remaining functions assigned to the language by any group (…).

#### Heritage

There are no remaining L1 speakers, but there may be some emerging L2 speakers or the language may be used for symbolic and ceremonial purposes only.

### (iv) Functional domains

Initially, digital word processing was restricted to large organizations and printing presses, but with the spread of PCs, desktop publishing became available at the household level. Similarly, the function of making public announcements, until recently restricted to the village worthy, became available to individuals, who can post on bulletin boards or (micro)blog. Altogether, the digital age ushered in, or made more accessible, many forms of communication hitherto restricted to small elites, and this is undoubtedly one of its main attractions. But for a language to spread to these new or newly democratized functional areas, one generally needs a bit of software. (The main exception is cellphone usage, which we had to ignore in this study for lack of data.) To quantify software support we use a simple three-stage hierarchy, roughly analogous to the questions probing literacy status in EGIDS, see the Methods section.

### (v) Wikipedia

Since digital ascent means active use of the language in the digital realm, we need to identify *at least one* active online community that relies on the language as its primary means of communication. There may be small bulletin boards, mailing lists, Yahoo, or Google groups scattered around, but experience shows that Wikipedia is always among the very first active digital language communities, and can be safely used as an early indicator of some language actually crossing the digital divide. The reason is that children, as soon as they start using computers for anything beyond gaming, become aware of Wikipedia, which offers a highly supportive environment of like-minded users, and lets everyone pursue a goal, summarizing human knowledge, that many find not just attractive, but in fact instrumental for establishing their language and culture in the digital realm. To summarize a key result of this study in advance: *No wikipedia, no ascent.*

The need for creating a wikipedia is quite keenly felt in all digitally ascending languages. This is clearly demonstrated by the fact that currently there are 533 proposals in incubator stage, more than twice the number of actual wikipedias. In fact, the desire to get a working wikipedia off the ground is so strong as to incite efforts at gaming the ranking system used by wikipedia, which sorts the various language editions at http://meta.wikimedia.org/wiki/List_of_Wikipedias simply by number of articles. The most blatant of these Potemkin wikipedias is #37, Volapük, which is based almost entirely on machine-generated geographic entries such as Kitsemetsa *Kitsemetsa binon vilag in grafän: Lääne-Viru, in Lestiyän. Kitsemetsa topon videtü 58°55′ N e lunetü 26°19′ L.* ‘Kitsemetsa is a village in Lääne-Viru County, in Estonia. It is at at latitude 58°55′ N and longitude 26°19′ E.’ The Methods section discusses how the effects of such gaming can be removed.

## Materials

All our data come from public repositories accessed between June 2012 and March 2013. A consolidated version of our main data table, 8,426 rows by 92 columns, is available as File S1. Here we provide only a brief overview of the main data sources, see File S2 for further details. The data is intended to cover the entire population of the world’s languages – some lacunae may remain, but internal consistency checks suggest that our coverage is over 95%.

The primary registry of data about the world’s languages, now charged with maintaining the ISO 639 standard for language codes, is the Ethnologue database of the Summer Institute of Linguistics (SIL International), see http://www.ethnologue.com. The latest (2012/02/28) publicly available version of the database distinguishes 7,776 languages, among them 376 that died since 1950 when SIL started to maintain the list.

We consulted several other sources, and our own dataset is larger by about 10% for the following reasons. First, we didn’t discard ancient/reconstructed languages such as Classical Chinese or Proto-Indo-European and artificial/constructed languages like Peano’s Interlingua (Latin Sine Flexione), which are by design out of scope for the Ethnologue. Second, our sources cover several languages that have only been recently discovered and have not yet completed the registry process: an example would be Bagata, a language spoken by one of the Scheduled Tribes in Andhra Pradesh. Third, we considered language groupings with online activity like Akan and Bihari irrespective of whether they meet the SIL criteria for ‘macrolanguage’. Whenever we encountered languages with no ISO code, and no code on the Linguist List (see http://linguistlist.org), we generated a non-authoritative internal code that begins with xx so as to maintain unique identifiers suitable for joining rows from different sources. For less commonly taught languages, we generally mention the ISO code (three lowercase letters) because the language names themselves are often subject to considerable spelling variation. Altogether, we have 7,879 ISO codes (the number is larger than the size of the February 2012 dump because the site now provides codes for many newly registered languages), with the balance coming from other sources, to which we now turn.

Perhaps the best organized of these is the Open Language Archives Community, ‘an international partnership of institutions and individuals who are creating a worldwide virtual library of language resources’, see http://www.language-archives.org. OLAC has some data for 7,478 of the 7,776 languages with ISO codes. Neither OLAC nor Wikipedia will consider languages without ISO code, so the lack of ISO status could in principle be a handicap for digital ascent. In practice, however, our conclusions can only be strengthened by the inclusion of these unregistered languages since they are already at the margin, with EGIDS level 6b or worse, while failure to ascend affects many languages at EGIDS level 4 or even better.

The last source aiming at encyclopedic completeness is the Endangered Languages Project hosted at http://www.endangeredlanguages.com which consolidates data from the Catalogue of Endangered Languages (ELCat), produced by the University of Hawai’i at Manoa, and The Institute for Language Information and Technology (The Linguist List) at Eastern Michigan University. We accessed the database on 2013/03/15, when it contained data for 3,175 languages. ELP uses a different scale of vitality, with categories critically endangered; severely endangered; endangered; threatened; and vulnerable, which correlate well with the higher EGIDS categories but are independently assessed. Since ELP considers vital languages (which are generally EGIDS 6a or less) out of scope, the fact that a language has no ELP page is generally a good sign. with

Less encyclopedic, but very relevant to our purposes, is the website of the Crúbadán Project, see http://borel.slu.edu/crubadan, which collects language data for endangered languages on the web. Version 2 covered 1,322 languages 2013/03/15 when we accessed the data, Version 1 started with 1,003 in 2006. The Crúbadán Project, quite independent from us, but consistent with our methodology, chose not to harvest material from closed archives such as the Rosetta Project (see http://rosettaproject.org) or metainformation such as the grammatical features collected in The World Atlas of Language Structures (see http://wals.info), since these are in no way indicative of digital use by native speakers.

Another highly relevant website is Omniglot, ‘the online encyclopedia of writing systems and languages’, see http://www.omniglot.com. Literacy in the traditional sense is a clear prerequisite of digital literacy, and languages without mature writing systems are unlikely to digitally ascend. Note that there are only 696 languages listed in Omniglot, and many of these are ancient or constructed languages without a live community. Even more relevant to our purposes is the level of support for computer-mediated activity in a given language. Here our basic data comes from inspecting Microsoft and Apple products for two levels of language support: *input* and *OS*. Input-level support means the availability of some specific method, such as Kotoeri for Japanese, to enter text in the writing system used for the language. Without an input method, digital ascent is impossible, but the converse unfortunately does not hold: the existence of some input method by no means guarantees an easy way to create text in the language, let alone vigorous digital language use. OS-level support means that all interaction conveyed by the operating system, such as text in dropdown menus or error messages, are provided in the language in question.

There are many languages with standard input methods but no standardized orthography, and the next step up the digital ladder is a spellchecker. The Crúbadán Project also considers this a relevant factor, and lists explicitly whether a Free/Libre Open Source Software (FLOSS) spellchecker exists. We also looked at HunSpell (the largest family of FLOSS spellcheckers, see [20]) for each language, and assessed its coverage by computing the percentage of words it recognizes in the wikipedia dump. Any number below 50% indicates the spellchecker is not mature.

Standardized orthography enables not just collective works like Wikipedia, itself an important indicator of digital vitality, but also the creation of larger documents. Again, the Crúbadán Project considers this a relevant factor, and lists whether the Bible and the Universal Declaration of Human Rights (UDHR) are available online. Collecting larger corpora, the lifeblood of modern language technology efforts, also requires standardized spelling. The relationship of digital language vitality and more sophisticated tools of modern computational linguistics such as parsers, speech and optical character recognition software, information extraction, and machine translation tools will be discussed in the next section.

## Methods

The EGIDS scale already comes with a clear notion of ascent, from oral use only (category 6) to acquiring literacy (5) and ‘vigorous oral use (…) reinforced by sustainable literacy’ (4). Further steps up the traditional scale are predicated on the level of (official) use: ‘used in work and mass media without official status to transcend language differences across a region’ (3); ‘used in education, work, mass media, and government within officially recognized regions of a nation’ (2); ‘used in education, work, mass media, and government at the nationwide level’ (1); and ‘widely used between nations in trade, knowledge exchange, and international policy’ (0) [21]. In the digital realm, it is also literacy that provides the pivotal step, and we begin by describing the main stages of acquiring it.

Stage one is some kind of *locale* or *i18n* (computer shorthand for ‘internationalization’) support that enables the input (writing) and output (reading) of native characters. On the whole the Unicode standard, already covering more than a hundred scripts and with a well-established mechanism for adding new ones, provides a solid basis for bringing any language to the digital age, as long as it is written (signed languages will be discussed separately). When a language is listed in Omniglot, we can assume it is past stage one. A weaker condition is the availability of online text in OLAC, a stronger condition would be the availability of an input method.

For the second stage we need a variety of word-level tools such as dictionaries, stemmers, and spellcheckers. Here support is more spotty – even the most broadly used tool, HunSpell [20], is available only for 129 languages, http://hlt.sztaki.hu/resources/hunspell. In spite of the uneven coverage and quality of these tools, they already represent a level of maturity that is very hard to match by an underresourced language. This is because spellcheckers enforce the unified literary standard of a koiné, with significant suppression of individual and dialectal variation. This stage was reached by English only in the 15th century (primarily as a result of the efforts of William Caxton), and many of the languages discussed here have neither undergone the painful process of koiné formation driven by internal needs nor want it to be imposed on them externally [22].

The third stage requires phrase- and sentence-level tools that can only be built on some preexisting character- and word-level standard, such as part-of-speech taggers, named entity recognizers, chunkers, speech recognition, and machine translation. In the tables presented at http://www.meta-net.eu/whitepapers/key-results-and-cross-language-comparison not even English has ‘Excellent’ support in these higher areas, which are key to avoiding long-term function loss. We surveyed Google Translate to probe this increasingly important area of functionality, but we emphasize here that stage three has more to do with the line between our top two categories, thriving (T) and vital (V), while our primary concern is with the gap between vital and still (S) languages. We have not surveyed speech and character recognition software, not because they are any less important, but because their quality still improves at a fast pace, and languages that lack these today may well acquire them in a hundred years.

Let us now describe the resolution of the classification system proposed here. In contrast to the 8 categories used in GIDS and the 13 used in EGIDS, we will identify only four classes of languages we call digitally **T**hriving, **V**ital, **H**eritage, and **S**till, roughly corresponding to the volume of active language use in the digital realm. Accordingly, the decision tree presented in Fig. 1 of [11] will be drastically simplified: we will have a major decision, whether a language is actively used in the digital realm, and two supplementary distinctions. The primary goal of our work is to investigate the dead/alive distinction in the digital domain, with the finer distinctions between degrees of ascent (vital versus thriving) and degrees of death (still versus heritage) seen as secondary.

**Figure 1:**
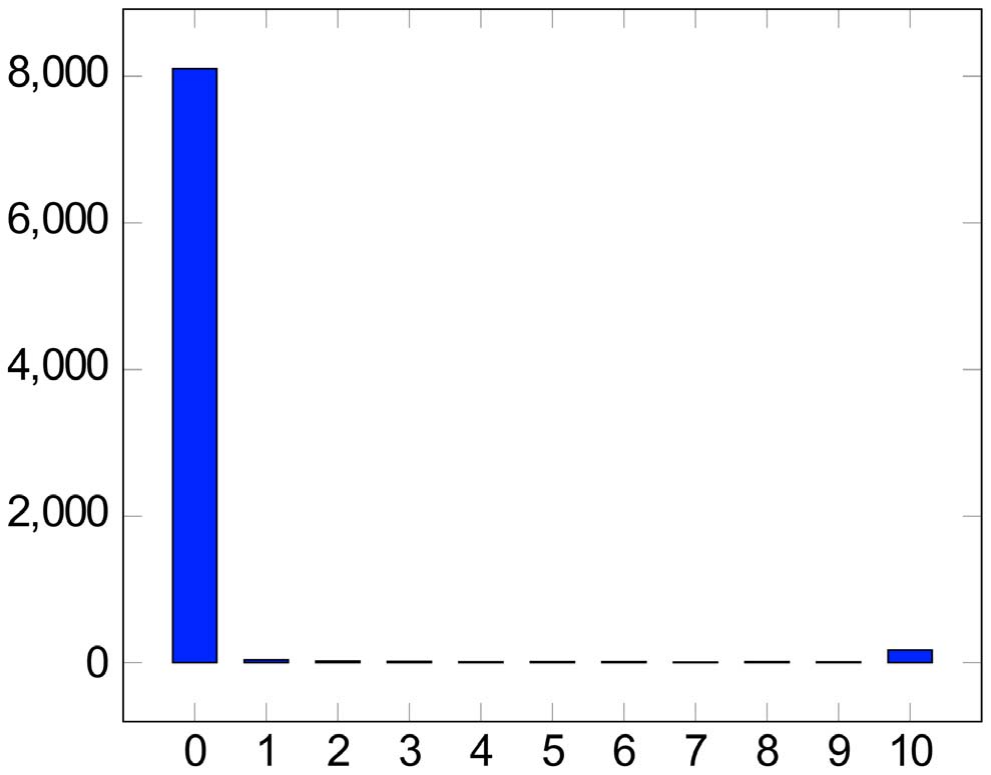
Bimodal distribution of two-way classifiers.

One possible method of fleshing out the classification would be to set some thresholds so that languages over 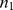 (say, 100,000) digital natives are considered thriving, those with fewer (but not zero) are considered vital, those with zero L1 speakers but more than 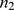 (say, 100) L2 speakers are considered heritage, and the rest still. While the method is commendably simple, it is rather arbitrary – why these 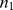 and 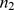, why not some other thresholds? Another problem is that it conflates the primary issue of digital ascent with the precise location of the cutoffs for the secondary distinctions – interesting as these may be on their own right, the key issue is the massive failure to digitally ascend, a failure whose dimensions, as we shall see, are quite independent of the choice of parameters.

The method we follow here allows for discovery: we take some clear, prototypical examples from each class, and use a standard machine learning technique, maximum entropy classification (multinomial logistic regression) [23], [24] to create a classifier that reproduces these seeds. Once the model is trained, we use it to classify the rest of the population. This way, not only the thresholds themselves, but the intrinsic error of threshold-based classification can be investigated based on the data. Further, we can check the effectiveness of the method both by internal criteria, such as the quality of the resulting classifier and its robustness under perturbation of the seeds, and by external criteria, such as comparison with other classification/clustering techniques.

Part of the simplification relative to EGIDS comes from the favorable demographics discussed above. For the traditional case, EGIDS makes an important distinction based on the last generation that has some proficient speakers: if these are the children, the language is threatened (category 6b); if the parents, the language is shifting (7); if the grandparents, it is moribund; and if the great-grandparents, it is nearly extinct (8b). In the digital case, once some speakers transition to the digital realm, their children and grandchildren automatically do so, and we feel justified in collapsing the higher numbers in EGIDS in a single category S. We also feel justified in collapsing the lowest numbers, 0 to 3, in a single category T, in that the questions EGIDS probes, whether a language has international, national, or regional scope, and whether it is official, make less sense in the digital realm that is by design international and unofficial.

As the examples of Classical Chinese, Sanskrit, or Latin show, even extinct languages can be digitally better resourced than many in the traditional sense thriving, but digitally impoverished languages. We will use the H category to account for those languages that are digitally archived, but not used for communication by native speakers. Their digital presence is *read only*, maintained by scholars. Wikipedia is supportive of heritage maintenance, but newly created wikipedias of extinct languages go to Wikia (the old ones are grandfathered and stay on Wikipedia proper). Since digital archives are here to stay, once a language has acquired heritage status it cannot lose it, and the global tide of digitization will hopefully move many languages from the still (lacking detectable digital presence) to the heritage (detectable but read-only digital presence) category. This movement, however, should not be mistaken for actual vitalization – as far as actual two-way communication in the language is concerned, both categories are digitally dead. The classical studies of language death lay down one absolutely unbreakable rule: *no community, no survival*. As Darwin, quoting Lyell, already notes “A language, like a species, when once extinct, never (…) reappears.” Modern Hebrew, a language viable both in the standard and in the digital sense, does not constitute a counterexample, inasmuch as neither its vocabulary nor its structure comes close to that of medieval Hebrew. As a matter of fact, new languages can be produced by children from unstructured input in a single generation [25] [26], but Modern Hebrew is best viewed as a representative of the main path of new language emergence, creole formation [27] [28].

Unlike heritage languages, which remain largely hidden from the non-specialist, vital languages are trivial to find – every computer user relies on one. Generally we can find billions of words of content in T and V languages, with millions of new words added every day. At the high end of digital ascent, thriving languages are used by very significant communities of both native (L1) and foreign (L2) speakers. There is a straightforward implicational hierarchy within the group of well resourced languages: if a language has OS-level support by Apple, it also has input-level support by Apple, a Microsoft language pack, and a FLOSS spellchecker. If it has input-level support by Apple, with 87% probability it will have input-level support by Microsoft as well, but if it lacks input-level support by Apple, Microsoft will remedy this with less than 1% probability. Similarly, languages with some input support either from Microsoft or Apple (or both) will have a spellchecker with 68% probability, while languages that lack input support have only 1.1% chance to have a spellchecker. Therefore it makes sense to simply count these resources and use the resulting number 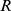 as a figure of merit: what we find is that there are only 244 languages that have 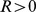. Of these, about a hundred are unquestionably viable.

We established the initial training seeds as follows. Those 16 languages that have the maximum 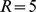 were collected in 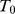: English, Japanese, French, German, Spanish, Italian, Portuguese (both Brazilian and European), Dutch, Swedish, Norwegian (Bokmål), Danish, Finnish, Russian, Polish, Chinese (both Traditional and Simplified), and Korean. Because of the implicational hierarchy noted above, these are exactly the languages with OS-level support by Apple, and this particular choice can be seen as reflective of criterion (ii), given the prestige role that Apple products now enjoy in the digital ecosystem. As we shall see, basing the choice on criterion (v), and selecting the top 16 wikipedia languages as alternate seed 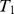 will lead to essentially the same results. Yet another reasonable criterion would be to consider the main competitors of under-resourced languages. From [16], where such competitors are called ‘polluters’, we see that the most important ones are English, Spanish, French, Russian, Italian, German, Dutch, and Portuguese, in this order, with other languages like Arabic or Polish listed as competitors only on one occasion. Again, we could use this set as an alternate T seed, and again the results would be unchanged.

Next we manually selected 84 languages which were unquestionably vital. From these, we randomly took two disjoint seeds 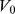 (40 languages) and 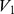 (40 languages). Typical examples included Banjar (bjn), Slovak (slk), Guaran (gug), Assamese (asm), Belarusian (bel), Kyrgyz (kir), Chichewa (nya), Armenian (hye), Hausa (hau), and Latvian (lvs). To establish the seed 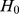 for the heritage group we manually selected a small group of unambiguous heritage languages: Aramaic (arc), Old Church Slavonic (chu), Coptic (cop), Manx (glv), Ancient Hebrew (hbo), Classical Chinese (lzh), Sanskrit (san), and Syriac (syc). An alternate seed 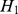, composed of Old English (ang), Avestan (ave), Cornish (cor), Geez (gez), Latin (lat), Mandaic (myz), Pali (pli), Classical Armenian (xcl), and Anglo-Norman (xno), was again selected to be disjoint from 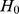. As with vital languages, we steered clear of the decision boundary, picking only very clear examples for the seed. In the Discussion section we provide several examples of the kind of languages like Ancient Greek (grc) that were not included in the seeds for building the classifiers, but were nevertheless deemed heritage by almost all classifiers.

At the other end of the digital divide, we selected those languages that have no wikipedia (not even an incubator), no UDHR, no Bible, no spellchecker, no Apple or Microsoft support at any level, no mention in Omniglot, and no data collected by the Crúbadán Project. This is not to say that such languages have no active digital presence at all, just that the best effort to find some, the Crúbadán Project, has failed to detect any. From this set of 6,541 languages we randomly took two small, disjoint training seeds, 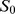 and 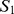, 75 languages each, for our still class. Typical examples are Rerau (rea), Terik (tec), East Limba (lma), Naami (bzv), Southern Puget Sound Salish (slh), Abure (abu), Lavukaleve (lvk), Tarao (tro), Korupun-Sela (kpq), and Lachi (lbt).

Other than converting the nominal classifications to numeral (e.g. EGIDS class 6a ‘vigorous’ to 6.0; 6b ‘threatened’ to 6.5; and 7 ‘shifting’ to 7.0) and applying a log transform to those fields (such as number of speakers or wikipedia size) that cover many orders of magnitude, we performed only two nontrivial data transformations. First, to control for the fact that the same number of (multibyte) characters will contain different amounts of information depending on writing system, we computed the character entropy of the language, and used it as a normalizing factor: for example, one Chinese character corresponds to about four Dutch characters, an effect quite visible if one compares the character counts of the same document, such as the UDHR or the Bible, in different languages. Second, in order to remove the effects of machine-generated wikipedia entries, we only considered those wikipedia pages to be ‘real’ that contain at least one paragraph with the equivalent of 450 German characters, pages that had less information were declared ‘fake’.

German was chosen as a baseline both because the German wikipedia is known to be high quality, and because before the adjustment it had the highest *real ratio*, defined as the number of ‘real’ pages divided by the total page count. After the adjustment it became clear that several wikipedias, such as Gujarati and Hebrew, have higher real ratios, but this does not affect our argument in that the same threshold could be expressed in Gujarati or Hebrew characters just as well. We define *adjusted wikipedia size* as the entropy-normalized total character count of real pages. The adjustment in most cases shrinks the wikipedia by less than a third, and in some cases such as Czech (real ratio 0.53) actually increases the size. Volapük, ranked 37 by article count, is ranked 163rd by adjusted wikipedia size.

## Results

Based on the four seeds 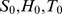, and 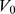 we trained several maximum entropy classifiers: 4-way classifiers S-H-V-T that distinguish all four classes; 3-way classifiers S-H-VT that treat T and V as one class of digitally alive languages but keep H and S separate; 3-way classifiers SH-V-T that treat S and H as one class of digitally dead languages but keep V and T separate; and 2-way classifiers SH-VT that simply probe the main digital divide, with T and V in one class, and H and S in the other.

Preliminary results of the classification were disappointing, only about 40% correct, as tested by 10-fold crossvalidation. However, as soon as we realized that some parameters like L1 and L2 span many orders of magnitude, and switched to logarithms for these as discussed in Methods (for a complete list, see File S2), classification performance improved markedly, with results now in the 85–100% range (see Table 1). Since random performance would be about 50% in a 2-way classification task, the fact that the 2-way results are in the 95–100% range already shows that the classes were established in a coherent fashion. It is evident from Table 1 that the 3-way task obtained by merging the live languages is easier than the 3-way task obtained by merging the dead languages.

**Table 1:** Classification accuracy (10-fold crossvalidation).

	Seed 0				Seed 1			
# feat	SH-VT	S-H-VT	SH-V-T	S-H-V-T	SH-VT	S-H-VT	SH-V-T	S-H-V-T
33	95.0	99.3	92.3	90.7	99.3	98.6	94.3	87.9
18	97.2	99.3	91.4	96.4	99.3	98.6	95.0	89.3
10	97.9	99.3	92.9	95.7	100.0	99.3	93.6	90.0
8	97.1	99.3	92.9	97.1	100.0	96.4	94.3	85.7
6	97.1	99.3	92.1	93.6	100.0	96.4	95.7	89.3

Maxent models are defined by feature weights. Those features that contribute little to the classification have small weights (in absolute value), those that contribute a lot have greater values. Remarkably, the performance of our classifiers, originally built on 33 features (for a complete list see File S2) improves markedly if we drop out those features that contribute little and retrain on the rest. Automated feature selection is a standard technique in machine learning, where it is used mostly to improve training speeds and generalization [29]. Here it has the further advantage of defending the system from a charge of arbitrariness: why did we use the Crúbadán definition of FLOSS spellchecker rather than the HunSpell list? The answer is that it doesn’t matter, since feature selection will automatically decide which, if any, of these will be used.

Unsurprisingly, the best predictor of digital status was the traditional status. The feature encoding the EGIDS assessments by SIL experts was selected in all models, the feature encoding the Endangered Languages Project assessments was selected in all but one. The next best set of features indicated the quality of the wikipedia, followed by the number of L1 speakers, the size of the Crúbadán crawl, the existence of FLOSS spellcheckers, and the number of online texts listed in OLAC. This last feature, currently our best proxy for the intensity of the heritage conservation effort, has been selected in less than 5% of the cases, and when selected, has only 20% of the weight of the leading feature on average, clearly demonstrating that conservation has negligible impact on digital ascent.

One question that can be raised about the classification we obtain is whether we have biased the results in any way by selecting the seeds the way we did. In regards to thriving languages, there is really very little freedom: clearly English, the FIGS languages (French, Italian, German, Spanish), the CJK languages (Chinese, Japanese, Korean), and the main languages of former colonial empires (Dutch, Russian, Portuguese), will come at the top of the vitality scale no matter how we look at the matter (these acronym groupings are widely used in natural language engineering). But for the rest, we could choose alternate seeds for heritage, still, and vital languages that were entirely disjoint from the initial set, yet obtain classifiers that are, for most purposes, identical to each other: in particular, the best SH-VT classifiers, which only use six features, correlate with each other with 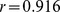. If we vote together the 10 best classifiers, a technique known as ‘bagging’ in the machine learning literature [30], one for each size considered for each seed set, as listed in the 2nd and 6th columns of Table 1, and give as many points to a language as there were classifiers that took it to be vital, we obtain the following distribution:

The distribution is sharply bimodal, with only 1.7% of the data in the middle, but this is to be expected from votes obtained from classifiers built to detect the same classes. The classifiers, both individually and collectively, identify a vast class of digitally dead languages that subsume over 96% of our entire data.

We emphasize that this massive die-off is not some future event that could, by some clever policies, be avoided or significantly mitigated – the deed is already done. We have identified a small group of about 170 languages (2%) that are ascending, or have already ascended, to the digital realm, and perhaps there is some hope for the 140 ‘borderline’ languages (1.7%) in the middle, a matter we shall discuss in the concluding section.

## Discussion

While the sheer magnitude of the failure to ascend is clear from the preceding, it would make no sense to declare some borderline language vital or still based on the result of any single classifier. Such individual judgment could only be made based on specific facts about the language in question, facts that need not be encoded in our dataset, and we see many examples of languages whose digital future is unwritten. That said, we can still demonstrate that the overall picture is remarkably robust under changes to the details of our method.

Because vital languages already have their survival assured, while heritage preservation is still very much an uphill battle, we looked more closely at 3-way classifiers that distinguishes heritage from still, but not thriving from vital. The best S-H-VT models discussed so far utilize 6–8 features, and have a precision of 97.1–100% based on 10-fold crossvalidation. To test robustness we randomized seed selection in the following manner.

We run two hundred paired experiments. For the first hundred 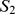 seeds we randomly take 75 languages from the group of 6,541 languages with no detectable live online presence, and another 75 for 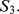 For 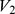 we take 40 from the 83 unambiguously vital languages collected in 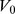, and use another 40 for 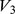. The 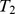 and 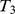 seeds are defined by taking the top 16 software support and the top 16 wikipedia languages – these seeds overlap in 13. The 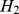 and 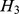 seeds will overlap completely, as we use the union of our earlier 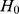 and 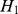. Thus, each classifier pair is built on 148 languages, of which only 20.3%, the heritage class and the bulk of the thriving, are shared across the pair. We chose this method to avoid any appearance of bias, since the heritage status of the languages we listed above with 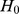 and 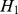 is hardly debatable, while many languages like Scots or Yiddish that would fall in the heritage class based on the vote of the first stage classifiers will still have strongly identified users who will, perhaps, dispute the classification the models provide.

When we look at the resulting S-H-VT-2 and S-H-VT-3 classifiers at 8 dimensions, there are small differences not just in the numerical parameters, but also in the dimensions selected: for example, some classifiers consider it relevant whether the language has an incubator wikipedia, while others ignore this factor and rely on the log number of L2 speakers instead. Nor are the classifiers perfect: internal testing (10-fold crossvalidation) shows accuracies of 95.8% on the average, with 2.1% variance. But when all is said and done, all these classifiers are highly correlated: binary classifiers built on the same pairs of seeds correlate with each other to 0.889
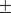
0.04. As a further check, in another hundred paired experiments we eliminated overlap completely. The resulting classifiers have very small heritage and thriving seeds, but the paired classifiers still correlate 0.823
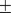
0.088, remarkably high considering that these pairs don’t share any training examples between them.

The first 200 classifiers, using 80% disjoint seeds (with the commonalities restricted to the unambiguous thriving and heritage cases as described above) estimate the digitally dead class to contain 8,049
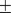
36 languages. The second 200, using completely disjoint seeds, shifts this number to 8,008
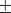
69. These classifiers, having been built on smaller seeds, are less reliable, but the overall picture is the same. No matter how we look at it, we have over 8,000 digitally dead languages, a quarter more than the 6,541 with no detectable online presence that we started out with. We estimate the size of the heritage subclass of the dead class by the same method to be 289
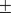
308, and the size of the digitally vital class (including the thriving languages) as 377
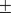
36, and will add one sigma to speak, rather optimistically, about 420 survivors. Altogether we estimate the rate of extinction to be 95.5%, with an uncertainty of about 0.4%.

**Figure 2:**
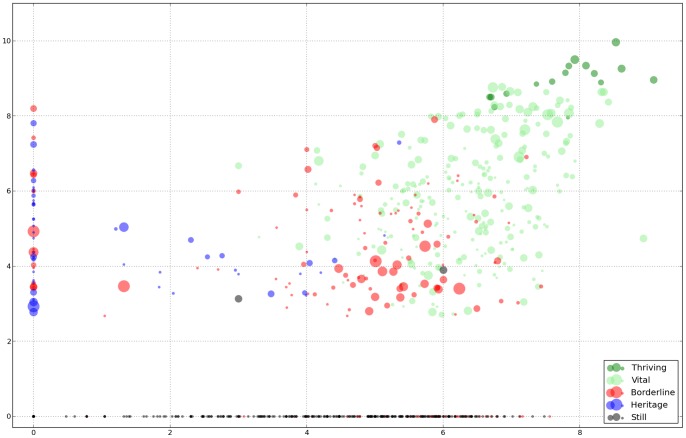
Adjusted wikipedia size plotted against number of speakers, log-log scales. Dot size shows real ratio, color shows status: **T**hriving dark green; **V**ital light green; **H**eritage blue; **S**till black; **B**orderline red. See main text for definitions, File S1 for underlying data.

For the following Figure 2 we selected a typical S-H-VT classifier pair, which has Spearman (rank) correlation 0.853 (Pearson correlation on a 0–1–2 scale would be 0.906). Whenever the two classifiers agreed, we used this result. We treat the 162 languages on which these disagree as members of an ad hoc ‘B’ (borderline) class, and break out the original 16 thriving languages from the VT (ascended) class, so that we can report results separately on vital and thriving. We plot only wikipedia (including incubator) languages. The 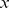 axis (log scaled) gives the number of speakers (plus one, so as not to make dead languages fall off the scale). The 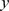 axis, also log scaled, shows the adjusted wikipedia size. The diameter of the dot is proportional to the real ratio defined at the end of the Methods section.

The 16 thriving languages, plotted in dark green on Fig. 2, have their digital future assured, at least on a hundred year scale – clearly wherever humanity goes these languages will go with them. The average number of native speakers in this group is 174.4 m, the real ratio is 0.34
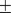
0.10, and the average adjusted wikipedia size is 1.63 g chars. Note that one of the six languages that receive the best (0) EGIDS rating, Arabic, while clearly digitally vital, has not reached thriving status yet, since Apple did not offer OS-level support at the time we collected the data and the Arabic wikipedia is still not in the top twenty.

Of the 252 additional languages classified vital, plotted in light green, only the original 83 forming the 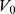 and 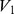 seeds have unambiguously vigorous language use, manifested in a significant digital community that generates millions of words of online material per year – the rest are largely borderline. Experience with the individual cases suggests that no more than 150 of these languages are actually vital but, in keeping with the conservative methodology outlined at the beginning, we are prepared to overestimate the vitality of the rest. The average number of native speakers in this group is 15.9 m, the real ratio is 0.22
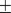
0.18, and the average adjusted wikipedia size is 32.5 m chars. While there is work to be done to make these languages truly thrive in the digital realm (for example, Hungarian is supported by Microsoft Word on the PC, but not on the Mac), we have little doubt that the rising tide of digitization will, in the next hundred years, carry at least half of them, hopefully even more. This group contains about two thirds (66%) of the EGIDS 1 languages; less than half (46%) of EGIDS 2; 13% of EGIDS 3; 8% of EGIDS 4; 2% of EGIDS 5; and less than 1% of all higher classes, for an average EGIDS of 3.3.

We emphasize that the 162 borderline languages, plotted in red, are not classifed ‘borderline’ but rather indicate the uncertainties inherent in the classification. The statistical summaries in Table 2 include these as well for the sake of completeness, but are not explained here, as these pertain to the margins rather than to true class averages.

**Table 2:** Summary characteristics of languages by class.

class	lang	r/t	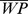	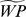	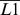	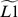	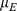	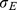
**T**	16	0.336	1630.9	877.4	174.4 m	67.4 m	0.69	0.46
**V**	252	0.225	32.5	0.74	15.9 m	3.1 m	3.29	1.98
**B**	162	0.148	2.17	0.003	1.39 m	0.1 m	5.73	1.90
**H**	51	0.144	2.25	0.018	8.79 k	0	7.83	0.93
**S**	307	0.003	0.00003	0	695 k	30 k	6.04	1.51

Class: **T**hriving, **V**ital, **B**orderline, **H**eritage, **S**till. lang: number of languages in class. r/t: ratio of real to total number of pages. 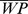: average adjusted Wikipedia size (millions of characters, entropy adjusted). 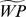: median adjusted Wikipedia size (millions of characters, entropy-adjusted). 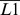: average number of native speakers. 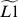: median number of native speakers. 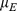: average EGIDS rating. 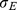 variance of EGIDS rating.

There are 51 heritage languages with Wikipedias, plotted in blue. Most of these wikipedias are grandfathered, because they were established before the current policy of banning dead languages was established, and it is likely that other heritage projects such as Classical Greek will eventually find a home on Wikia (as opposed to wikipedia.org). The average number of speakers is 8,787 (because several languages like Breton and Proven cal are listed with significant numbers of L1 speakers in the Ethnologue), the real ratio is 0.10
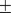
0.12 (we consider any real ratio above.1 reasonable), and the adjusted wikipedia size is 2.25 m chars. The large EGIDS average, 7.83, is quite reflective of their heritage status. Typical examples (as found by the classifiers, as opposed to the manually selected seeds listed in Methods) include Cree (cre), Dalmatian (dlm), Middle Dutch (dum), Ido (ido), Gothic (got), Old Norse (non), Pipil (ppl), Old Prussian (prg), Romagnol (rgn), and Samogitian (sgs).

There are 307 still languages, plotted in black, where no digital natives can be raised. The average number of speakers is 0.7 m, still quite sizeable, but the wikipedias are mostly incubators, essentially empty after adjustment. A typical example is Kanuri (kau), with main dialects Tumari (krt), Manga (kby), and Beriberi (knc), with EGIDS status 6a, 5, and 3 respectively. With vigorous language use, radio and TV broadcasts in the language, and a total of 3.76 m speakers, the language, at least the Central (Beriberi) dialect, is not on anybody’s radar as endangered – to the contrary, there are only 337 languages with EGIDS 3 or better. Yet the wikipedia was closed for lack of native language content and community, and the Crúbadán crawl listing three documents for less than 5,000 words total. The average EGIDS rating is 6.04, and the majority of the world’s languages are within one sigma of this value, consistent with our assessment that the majority of the world’s languages are digitally still.

## Conclusions

We have machine classified the world’s languages as digitally ascending (including all vital, thriving, and borderline cases) or not, and concluded, optimistically, that the former class is *at best* 5% of the latter. Broken down to individual languages and language groups the situation is quite complex and does not lend itself to a straightforward summary. In our subjective estimate, no more than a third of the incubator languages will make the transition to the digital age. As the example of the erstwhile Klingon wikipedia (now hosted on Wikia) shows, a group of enthusiasts can do wonders, but it cannot create a genuine community. The wikipedia language policy, https://meta.wikimedia.org/wiki/Language_proposal_policy, demanding that “at least five active users must edit that language regularly before a test project will be considered successful” can hardly be more lenient, but the actual bar is much higher. Wikipedia is a good place for digitally-minded speakers to congregate, but the natural outcome of these efforts is a heritage project, not a live community.

A community of wikipedia editors that work together to anchor to the web the culture carried by the language is a necessary but insufficient condition of true survival. By definition, digital ascent requires use in a broad variety of digital contexts. This is not to deny the value of heritage preservation, for the importance of such projects can hardly be overstated, but language survival in the digital age is essentially closed off to local language varieties whose speakers have at the time of the Industrial Revolution already ceded both prestige and core areas of functionality to the leading standard koinés, the varieties we call, without qualification, French, German, and Italian today.

A typical example is Piedmontese, still spoken by some 2–3 m people in the Torino region, and even recognized as having official status by the regional administration of Piedmont, but without any significant digital presence. More closed communities perhaps have a better chance: Faroese, with less than 50 k speakers, but with a high quality wikipedia, could be an example. There are glimmers of hope, for example [2] reported 40,000 downloads for a smartphone app to learn West Flemish dialect words and expressions, but on the whole, the chances of digital survival for those languages that participate in widespread bilingualism with a thriving alternative, in particular the chances of any minority language of the British Isles, are rather slim.

In rare cases, such as that of Kurdish, we may see the emergence of a digital koiné in a situation where today separate Northern (Kurmanji), Central (Sorani), and Southern (Kermanshahi) versions are maintained (the latter as an incubator). But there is no royal road to the digital age. While our study is synchronic only, the diachronic path to literacy and digital literacy is well understood: it takes a Caxton, or at any rate a significant publishing infrastructure, to enforce a standard, and it takes many years of formal education and a concentrated effort on the part of the community to train computational linguists who can develop the necessary tools, from transliterators (such as already powering the Chinese wikipedia) to spellcheckers and machine translation for their language. Perhaps the most remarkable example of this is Basque, which enjoys the benefits of a far-sighted EU language policy, but such success stories are hardly, if at all, relevant to economically more blighted regions with greater language diversity.

The machine translation services offered by Google are an increasingly important driver of cross-language communication. As expected, the first several releases stayed entirely in the thriving zone, and to this day all language pairs are across vital and thriving languages, with the exception of French – Haitian Creole. Were it not for the special attention DARPA, one of the main sponsors of machine translation, devoted to Haitian Creole, it is dubious we would have any MT aimed at this language. There is no reason whatsoever to suppose the Haitian government would have, or even could have, sponsored a similar effort [32]. Be it as it may, Google Translate for any language pair currently likes to have gigaword corpora in the source and target languages and about a million words of parallel text. For vital languages this is not a hard barrier to cross. We can generally put together a gigaword corpus just by crawling the web, and the standardly translated texts form a solid basis for putting together a parallel corpus [33]. But for borderline languages this is a real problem, because online material is so thinly spread over the web that we need techniques specifically designed to find it [16], and even these techniques yield only a drop in the bucket: instead of the gigaword monolingual corpora that we would need, the average language has only a few thousand words in the Crúbadán crawl. To make matters worse, the results of this crawl are not available to the public for fear of copyright infringement, yet in the digital age what cannot be downloaded does not exist.

The digital situation is far worse than the consensus figure of 2,500 to 3,000 endangered languages would suggest. Even the most pessimistic survey [34] assumed that as many as 600 languages, 10% of the population, were safe, but reports from the field increasingly contradict this. For British Columbia, [35] writes:

Here in BC, for example, the prospect of the survival of the native languages is nil for all of the languages other than Slave and Cree, which are somewhat more viable because they are still being learned by children in a few remote communities outside of BC. The native-language-as-second-language programs are so bad that I have NEVER encountered a child who has acquired any sort of functional command (and I don’t mean fluency - I mean even simple conversational ability or the ability to read and understand a fairly simple paragraph or non-ritual bit of conversation) through such a program. I have said this publicly on several occasions, at meetings of native language teachers and so forth, and have never been contradicted. Even if these programs were greatly improved, we know, from e.g. the results of French instruction, to which oodles of resources are devoted, that we could not expect to produce speakers sufficiently fluent to marry each other, make babies, and bring them up speaking the languages. It is perfectly clear that the only hope of revitalizing these languages is true immersion, but there are only two such programs in the province and there is little prospect of any more. The upshot is that the only reasonable policy is: (a) to document the languages thoroughly, both for scientific purposes and in the hope that perhaps, at some future time, conditions will have changed and if the communities are still interested, they can perhaps be revived then; (b) to focus school programs on the written language as vehicle of culture, like Latin, Hebrew, Sanskrit, etc. and on language appreciation. Nonetheless, there is no systematic program of documentation and instructional efforts are aimed almost entirely at conversation.

Cree, with a population of 117,400 (2006), actually has a wikipedia at http://cr.wikipedia.org but the real ratio is only 0.02, suggestive of a hobbyist project rather than a true community, an impression further supported by the fact that the Cree wikipedia has gathered less than 60 articles in the past six years. Slave (3,500 speakers in 2006) is not even in the incubator stage. This is to be compared to the over 30 languages listed by the Summer Institute of Linguistics for BC. In reality, there are currently less than 250 digitally ascending languages worldwide, and about half of the borderline cases are like Moroccan Arabic (ary), low prestige spoken dialects of major languages whose signs of vitality really originate with the high prestige acrolect. This suggests that in the long run no more than a third of the borderline cases will become vital. One group of languages that is particularly hard hit are the 120+ signed languages currently in use. Aside from American Sign Language, which is slowly but steadily acquiring digital dictionary data and search algorithms [36], it is perhaps the emerging International Sign [37] that has the best chances of survival.

There could be another 20 spoken languages still in the wikipedia incubator stage or even before that stage that may make it, but every one of these will be an uphill struggle. Of the 7,000 languages still alive, perhaps 2,500 will survive, in the classical sense, for another century. With only 250 digital survivors, all others must inevitably drift towards digital heritage status (Nynorsk) or digital extinction (Mandinka). This makes language preservation projects such as http://www.endangeredlanguages.com even more important. To quote from [6]:

Each language reflects a unique world-view and culture complex, mirroring the manner in which a speech community has resolved its problems in dealing with the world, and has formulated its thinking, its system of philosophy and understanding of the world around it. In this, each language is the means of expression of the intangible cultural heritage of people, and it remains a reflection of this culture for some time even after the culture which underlies it decays and crumbles, often under the impact of an intrusive, powerful, usually metropolitan, different culture. However, with the death and disappearance of such a language, an irreplaceable unit in our knowledge and understanding of human thought and world-view is lost forever.

Unfortunately, at a practical level heritage projects (including wikipedia incubators) are haphazard, with no systematic programs of documentation. Resources are often squandered, both in the EU and outside, on feel-good revitalization efforts that make no sense in light of the preexisting functional loss and economic incentives that work against language diversity [38].

Evidently, what we are witnessing is not just a massive die-off of the world’s languages, it is the final act of the Neolithic Revolution, with the urban agriculturalists moving on to a different, digital plane of existence, leaving the hunter-gatherers and nomad pastoralists behind. As an example, consider Komi, with two wikipedias corresponding to the two main varieties (Permyak, 94,000 speakers and Zyrian, 293,000 speakers), both with alarmingly low (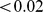) real ratios. Given that both varieties have several dialects, some already extinct and some clearly still, the best hope is for a koiné to emerge around the dialect of the main city, Syktyvkar. Once the orthography is standardized, the university (where the main language of education is Russian) can in principle turn out computational linguists ready to create a spellchecker, an essential first step toward digital literacy [39]. But the results will benefit the koiné speakers, and the low prestige rural Zyrian dialects are likely to be left behind.

What must be kept in mind is that the scenario described for Komi is optimistic. There are several hundred thousand speakers, still amounting to about a quarter of the local population. There is a university. There are strong economic incentives (oil, timber) to develop the region further. But for the 95% of the world’s languages where one or more of these drivers are missing, there is very little hope of crossing the digital divide.

## Supporting Information

File S1 Main data table.
(TSV)

File S2 Details on data sources and encoding in S1.
(PDF)
