# Supplementary material for: Digital Language Death
Source: PLoS One. 2013 Oct 22;8(10):e77056. doi: 10.1371/journal.pone.0077056 (PMC3805564; doi:10.1371/journal.pone.0077056)
Supplement: File S2 — Details on data sources and encoding in S1. (PDF) [file pone.0077056.s002.pdf]

Supplementary Material S2: Appendix

Supporting information S1, also accessible at `http://hlt.sztaki.hu/resources/dld-joined.tsv` used the latest (February 2012) Ethnologue dump, and May 2012 Wikipedia dumps except for the incubator projects, which multiplied rapidly at the time. The incubators at Wikimedia, OLAC, ELP, and the Crúbadán sites were crawled in March 2013. Software readiness reflects MacOS 10.6.8 and Windows 7, Google Translate was assessed December 2012. Features marked by **log** are incremented by one and log transformed before classification. Features marked by **ignore** were not used as input to the classification, they are presented only to help the readers search for particular languages. The abbreviations here are taken verbatim from the header of the main table.

1. unique_join_code – SIL or Linguist List code where available. For languages without clearly identifiable SIL code in ELP and Crúbadán non-authoritative codes beginning with `xxe` and `xxx` were generated **ignore**
2. wikiname – the two-letter (sometimes longer) code used in Wikipedia **ignore**
3. EthLanguageStatus – the EGIDS status of the language according to the Ethnologue. Values 6b and 8b were replaced by 6.5 and 8.5 so as to keep the other values of the scale intact. The Ethnologue is fairly complete: based on lack of overlap with other language lists we estimate its coverage on non-extinct non-artificial languages to be well above 90%. At this stage, non-threatened languages are highly unlikely to be added, so for such languages we supplied an EGIDS value of 7.7, the weighted average of the threatened classes
4. L1 – number of people speaking the language natively (L1 speakers) **log**
5. L2 – number of people speaking the language as second language (L2 speakers) **log**
6. MACinput – input-level support by Apple
7. MACsupp – OS-level support in MacOS 10.6.8
8. MSifpack – input-level support by Microsoft
9. MSpack – OS-level support in Windows 7.
10. TLDs – whether a national level Top Level Domain (not `.com, .org, .edu`) appeared in the top three domains that the crawl found language data in. Potentially a proxy for national-level organization in cyberspace
11. WPincubatornew – whether Wikipedia had an incubator for the language in March 2013
12. WPsizeinchars – raw (unadjusted) character count of wikipedia **log**
13. adjustedWPsize – character count of ‘real’ wikipedia pages, normalized for character entropy **log**
14. articles – number of articles in wikipedia **log**
15. realtotalratio – proportion r/t of ‘real’ wikipedia pages
16. avggoodpagelength – average length of ‘real’ wikipedia pages **log**
17. cru1Characters – number of characters found by the first Crúbadán crawl **log**
18. cru1Docs – number of documents found by the first Crúbadán crawl **log**
19. cru1FLOSSSplChk – whether a FLOSS spellchecker exists according to the first Crúbadán summary

20. cru1UDHR – whether a translation of the Universal Declaration of Human rights exists according to the first Crúbadán summary
21. cru1WT – whether an online Bible exists at `watchtower.org` according to the first Crúbadán summary
22. cru1Words – number of words found by the first Crúbadán crawl **log**
23. cru2Characters **log**, cru2Docs **log**, cru2FLOSSSplChk, cru2MSinput, cru2UDHR, cru2WT, cru2Words **log** – same as above, except data is taken from the second Crúbadán crawl.
24. endclass – classification according to the Endangered Languages Project: at risk: 4; vulnerable: 5; threatened: 6; endangered/unknown: 7; critically endangered: 8; severely endangered: 9. Since the project aims at completeness, languages it excludes are assigned to class 2
25. hunspellcoverage – the percentage coverage Hunspell has on the wikipedia dump
26. hunspellstatus – whether a HunSpell spellchecker exists for the language
27. inomni – whether the language is written in a script listed in Omniglot
28. laPrimarytextsOnline – the number of OLAC primary texts online **log**
29. seedstatus – training labels manually set to C, V, H, or M for languages in the respective seeds
30. cru2Classification – The more elaborate linguistic classification listed in Crúbadán **ignore**
31. PrintName – standardized English language name **ignore**
32. LanguageLocal – the local name of the language **ignore**
33. cru2NameNative – the local name of the language according to Crúbadán **ignore**
34. sample_result – the result of voting together two paired classifiers **ignore**
